# Toward an Integrative Approach to the Study of Positive-Affect-Related Aggression

**DOI:** 10.1177/17456916231200421

**Published:** 2023-11-08

**Authors:** Joyce Emma Quansah, Jean Gagnon

**Affiliations:** 1 Department of Psychology, University of Montreal; 2 Centre for Interdisciplinary Research in Rehabilitation of Greater Montreal, Montreal, Quebec, Canada; 3 Laboratoire d’Électrophysiologie en Neuroscience Sociale, University of Montreal

**Keywords:** aggression, violence, motivation, reward, intragroup processes, interpersonal processes, self

## Abstract

Research on aggression usually aims at gaining a better understanding of its more negative aspects, such as the role and effects of aversive social interactions, hostile cognitions, or negative affect. However, there are conditions under which an act of aggression can elicit a positive affective response, even among the most nonviolent of individuals. One might experience the “sweetness of revenge” on reacting aggressively to a betrayal or social rejection. A soldier may feel elated after “shooting to kill” in the name of the flag. There are many factors that contribute to the appeal of aggression, but despite growing interest in researching these phenomena, there is still no unitary framework that organizes existing theories and empirical findings and can be applied to a model to generate testable hypotheses. This article presents a narrative review of the literature on positive-affect-related forms of aggression and explores the role of aggression in eliciting positive affect across diverse social situations and relational contexts. An integrative model that unifies existing theories and findings is proposed, with the objective to inspire and inform future research.

Although most contemporary societies generally condemn aggression and violence and intuitively associate it with negative effects, there are many examples of aggressive acts that elicit a positive affective response. Evidence of a relationship between aggression and positive affect (PA)—a subjective experience of “pleasurable” emotions such as happiness, excitement, or joy—has reportedly been associated with a range of conditions and social contexts that manifest across ages and cultures (for examples, see Chester, 2017). Historical examples include 16th- and 17th-century recreational fighting in Renaissance Venice, whereby community members used sticks and stones to attack rival neighborhood members for fun (Ingle & Cross, 2004; Potegal, 2006). During 19th-century faction fights in Ireland, mass brawls were often met with collective enthusiasm (Potegal, 2006). Twentieth-century American loggers would happily volunteer to fight and, in doing so, improve their reputation, self-esteem, and personal status (Ingle & Cross, 2004). Research conducted in the townships of South Africa provide more modern-day examples, with gang-affiliated youth reporting a “high” from having committed brutal acts of violence and mutilations (Weierstall et al., 2013). Research on bar-related aggression suggests that, at least for some males, fist fights are often carried out purely for pleasure, fun, and excitement (Graham et al., 2011; Miller et al., 2014).

Although these examples reflect a more direct relationship between aggression and PA, the response can also be elicited indirectly—through vicarious forms of aggression (i.e., observing or hearing about aggressive conduct committed by others). Enjoyment of violence in sports, for example, is apparent in the cheers from the stands, as fans delight in seeing their hockey players throw off their gloves and fight with rival team players. The popularity of violent video games also points to the appeal of “virtual aggression,” with first-person shooter games among the best-selling in the world (Hartmann & Vorderer, 2010). Users often temporarily “forget” that their experience is not real, which facilitates total transportation and immersion into the enjoyment of shooting and killing other social beings (Atkinson & Rodgers, 2016; Sherry, 2004; Skalski et al., 2006).

This phenomenon, which we refer to as PA-related aggression, can therefore take many forms and be carried out in a variety of response modes (e.g., verbal, physical, direct, indirect, relational). It can result from reacting aggressively to an aversive or provocative social event (i.e., reactive aggression), or it can be pursued through unprovoked attacks on vulnerable victims (i.e., proactive aggression). It can emerge when interacting with friends, colleagues, or intimate partners (interpersonal aggression) or between one or several members of different social groups (intergroup aggression). The social context in which these interactions occur may provide insight into the role of aggression in eliciting PA. For example, we can imagine an interpersonal context in which a child who, being picked last by their peers for a sports team, becomes overwhelmed with negative emotions and thoughts. To repair their negative affective state (i.e., increase PA) and injured self-esteem, they react to the situation by lashing out at their peers with verbal attacks and name-calling. This immediately improves their mood and leaves them feeling satisfied and proud. Similarly, we can imagine an intergroup social context in which spectators at a sporting event who, having watched their team get a “beating” from their archrivals, experience a collective sense of disappointment and defeat. They collectively (and proactively) organize a postgame brawl, targeting and attacking rival spectators after the game. This collective violence is met with feelings of elation, excitement, and the “sweet satisfaction” of having successfully restored their team’s honor.

Given the variety of ways in which aggression becomes associated with PA, how can we identify specific areas to target for intervention? The “when” at which the PA occurs, or its duration, may appear as variables of interest; however, these temporal components of the PA-aggression link can vary considerably. For example, after social rejection, it is possible that—only once the child has restored their self-esteem and achieved a sense of satisfaction through aggression—they then feel an increase in PA. Alternatively, the simple act of anticipating the suffering of the offender may elicit a similar affective response, even before the aggression has occurred. The duration of these aggression-related PA experiences may depend on the perceived effectiveness of the act in helping achieve some goal (e.g., obtaining retribution, restoring self-esteem, showing dominance or power), but they may also depend on external outcomes that are unforeseen (e.g., getting “caught” after the act, experiencing postaggression guilt).

Another approach to identifying potential targets for intervention might involve taking a closer look at the role of aggression in driving increases of PA as it occurs during different types of social interactions as well as within different relational contexts. Having a better understanding of when and how an act that is predominantly considered objectively “bad,” and often results in devastating physical and psychological consequences (Berkowitz, 1993; Shaver & Mikulincer, 2011), becomes subjectively perceived as “good,” appears critically important. This may ultimately have profound implications for our ability to develop working hypotheses and generate ideas on how to intervene and reduce cycles of violence.

## Main Objectives of the Article

Several theoretical explanations have been proposed to explain the role of aggression in eliciting PA. With reactive forms, psychological processes related to emotion regulation mechanize aggression in pursuit of improving or repairing mood after an aversive social event such as social rejection. With more proactive or instrumental forms, such as “appetitive aggression,” a distinct biological preparedness has been posited to manifest as an innate desire to increase arousal and PA through extreme acts of violence (Elbert et al., 2017a). These PA-related aggressive interactions have been evidenced across different relational contexts, both interpersonal and intergroup (occurring between one or several members of different groups), with positive appraisals of aggressive behavior being linked to individual trait factors, psychological processes, and strong social influences.

Despite this growth in a somewhat neglected field of study, to our knowledge there is no model of PA-related aggression that unifies existing theories or findings. It is thus challenging to compare or replicate findings, or even distinguish PA-related aggression from other phenomena. Although several classification schemes exist for human aggression more generally, its distinct link with PA is found only in a handful of aggression “subtypes” (e.g., appetitive aggression, sadism, schadenfreude). This gap in the literature has led to puzzling inconsistencies and obscured our understanding of how aggression potentially elicits PA. This article presents an overview of the PA-related aggression literature and considers the many ways in which aggression manifests across a variety of social interactions. To address the need for a unitary construct, our second objective is to propose an integrative model that can reconcile conflicting observations in the literature and promote growth in the field.

## PA and Aggression: Definitions, Distinctions, and Constructs

What is PA? According to the dominant theories of emotion in psychology and affective neuroscience, *affect* is conceptualized as a subjective experience manifesting as a feeling, emotion, or mood (Hogg & Abrams, 2007). An affective experience or state is thought to emerge from the interaction of two main neurophysiological dimensions: valence (on a pleasure-displeasure continuum) and arousal (Hogg & Abrams, 2007; Posner et al., 2005). Valence refers to the subjective evaluation of the experience as being either positive (pleasant) or negative (aversive) in quality, whereas arousal (or alertness) pertains to the degree in which a stimulus or event engages the individual (Posner et al., 2005). For example, an experience that elicits PA can be described as a feeling of joy, contentment, or pleasure (positive valence), which may simultaneously be described as exciting or thrilling (high arousal) or evoke a sense of being relaxed or euphoric (low arousal). PA-related aggression is thus any manifestation of aggression that directly or indirectly elicits one or several of these positive subjective experiences.

Aggression is commonly described in the social-psychology literature as an observable behavior underlined by an incentive to cause harm to another person who intends to avoid that harm (Allen & Anderson, 2017; Bushman & Huesmann, 2010). Although this definition may prove useful for testing more traditional theories of aggression, in the context of PA-related aggression using such strict cutoffs would result in excluding the more indirect, at times vicarious, ways in which aggression elicits PA. For example, when using the “observable-behavior” criteria, we disregard the potential pleasure derived from watching aggressive media content or violent sports (Cikara, 2015; Cikara et al., 2011), or from thinking about committing an aggressive act (e.g., revenge rumination), or by enacting it virtually through violent video gaming. Indeed, incidents of PA-related aggression may not always be observable, or even actualized in the real world. There is also the risk that, in an experimental setting, participants fear being perceived as socially deviant or perverse when admitting that aggression can sometimes feel good. Many of the studies examining PA-related aggression in a laboratory will use vignettes and/or self-report measures (e.g., rating scales, questionnaires), which allows participants to reflect on and appraise their own personal experience. This can be done retroactively, with participants indicating what they thought or felt during a previous aggressive interaction, or prospectively, by indicating how they would feel if or when it happened in the future.

Although there are indeed limitations involved when using explicit measures to quantify subjective human experiences, these methods may mitigate the effects of social inhibitions (because of moral and/or cultural norms and expectations) and further minimize the risk of pre- or postaggression guilt. Examining these “nontraditional” forms of aggression can allow researchers access to some of the more discrete, hidden, or unexpressed manifestations of the phenomena. Ultimately, the association between aggression and PA can exist with or without an observable or behavioral enactment, and considering these nontraditional forms may ultimately inform a more nuanced understanding of this relationship.

## PA-Related Aggression: Organizing Findings

Given that most, if not all, forms of aggression have the potential to elicit PA, how can we distinguish PA-related aggression from more “traditional” forms? In the social-psychology literature, one way to organize aggressive episodes is to focus on the *response mode*, which entails making a distinction between whether the aggression is carried out physically, verbally, directly, indirectly, overtly, covertly, or relationally (Allen & Anderson, 2017). Aggression is also commonly classified using the notion of *instigation*, whereby a distinction is made between aggression that is reactive or retaliatory in nature from aggression that is proactive or unprovoked. In the context of PA-related aggression, a reactive example would involve an initial provocation event (e.g., a bullying episode, a breakup between romantic partners), an aggressive reaction to this event (e.g., physical altercation, indirect attack on social media) that increases—or is associated with an increase of—PA. A proactive example would occur without the initial (or immediate) provocation event (e.g., an online troll peruses online forums in search of an unsuspecting victim) that leads to some form of aggression (e.g., writing offensive and hurtful comments on their online-profile page) and an increase in PA.

Importantly, reactive and proactive aggression can (and often do) overlap, transform into other forms, or become cyclical (which we discuss later). For example, a person may initially be motivated to improve their mood by hurting someone who has provoked them (PA-related reactive aggression), but because the immediate situation fails to afford an opportunity to pursue this desire (e.g., because of security surveillance at a sporting event, or the risk of “getting in trouble” at school), they seek out opportunities to “feel good” by (proactively) aggressing others in other environments or situations (e.g., displaced aggression). Further, the underlying motivations driving PA-related aggression are not always clear-cut. A person may simultaneously be motivated to hurt a person to get revenge, improve their mood, and “look cool” in front of their peers. What is essentially distinct about PA-related aggression, above and beyond response mode, instigation status, or underlying motive, is that it characteristically results in eliciting or increasing PA.

## PA-Related Aggression: Supporting Evidence

There are many different terms used to refer to PA-related aggression in the social-psychology literature, including, for example, “pleasure,” “appetitive,” ‘rewarding,” “hedonia,” “hedonic,” “joy,” “bloodlust,” and “combat high” (Benenson et al., 2008; Chester, 2017; Eadeh et al., 2017; Elbert et al., 2017b; Golden & Shaham, 2018; Ramírez et al., 2005). There have also been conceptual models (or constructs) proposed, specific to the PA-aggression link, and each with its own theoretical perspective and mechanistic explanation of the relationship. A selection of the most developed models is shown in Table 1, which provides an overview of key characteristics, everyday examples, and supporting evidence for PA-related aggression. In the following sections, we review the literature on PA-related aggression using the common reactive/proactive distinction; we also review nontraditional forms. Throughout this discussion, we ask the reader to consider how aggression may serve as a vehicle to achieve goals in a variety of social contexts, both within interpersonal and intergroup relations and distinctly in its capacity to elicit PA.

**Table 1.** Characteristics, Examples, and Empirical Evidence of Forms of PA-Related Aggression

Constructs (and related concepts)	Definitions	Theoretical model	Examples	Supporting evidence
PA-related impulsive/reactive aggression (“sweet revenge,” catharsis)	An aggressive reaction to a perceived offense to improve/regulate mood (e.g., increase PA) and restore positive self-image	Emotion-regulation	An ex-employee feels a rush of happiness and satisfaction after slashing the tires of their former employer’s car	Carlsmith et al., (2008); Chester & DeWall (2017); Eadeh et al. (2017); Threadgill & Gable (2020)
Appetitive aggression (bloodlust, lust for violence, combat high)	An innate (biologically based) desire to perpetrate controlled, target-oriented acts of aggression for the sole purpose of experiencing violence-related pleasure or positive arousal	Evolution-based	A group of soldiers celebrate after shooting and killing an enemy troop during combat	Elbert et al. (2017a, 2017b, 2018); Moran et al. (2014); Weierstall et al. (2013)
Everyday sadism; sadism	Various forms of subclinical sadism prevalent in most modern societies and cultures; a tendency to derive pleasure from intentionally inflicting pain, suffering, or humiliation on others	Personality-based	An Internet troll takes pleasure in taunting the friends and family of a recently murdered classmate	Baumeister & Campbell (1999); Buckels et al. (2014); March & Steele (2020)
Schadenfreude	To derive joy (pleasure) on account of another person(s) suffering or misfortune	German-based concept (compound of *schaden*, meaning “to damage or harm,” and *freude*, meaning “joy”)	A boxing fan feels a thrilling rush of excitement when their favorite fighter knocks out their opponent	Cikara (2015); Dalakas et al. (2015); Leach et al. (2003)

Note: PA = positive affect.

### Reactive forms of PA-related aggression

During everyday social interactions, it is not uncommon for conflicts to arise that result in a person feeling hurt, angry, or rejected. It is also quite common to believe that “getting revenge” through aggression can effectively repair these negative emotional states (Bushman et al., 2001; Chester & DeWall, 2015). Across multiple studies with undergraduate student participants, Chester and DeWall (2017) tested the hypothesis that the desire to use aggression after social rejection is motivated by a personal desire to feel better. Results supported the notion that reacting to social rejection aggressively can indeed be driven by a desire to repair a negative mood, an effect that was mediated by personal expectations that harming the “rejector” would increase PA (Chester & DeWall, 2017). Further, when participants were given the opportunity to react aggressively after an experimental induction of social rejection (using a voodoo doll task following a cyberball paradigm), findings indicated that aggression successfully reduced negative affect (NA) and increased PA, as measured using self-reports. When comparing findings across similar studies (Bushman et al., 2001; Chester & DeWall, 2015), it appears that the strength of the personal belief that aggression can serve as a quick and effective means to improve mood moderates the strength of the PA-aggression link.

The role of reactive aggression in driving increases of PA after a social-rejection event is also supported by EEG evidence. Threadgill and Gable (2020) used a reward-related event-related potential (reward positivity, or RewP) and a reactive-aggression computer task to assess neural responses in the context of revenge. Results revealed that, when angry, participants reported greater excitement and showed larger reward positivity (i.e., greater RewP amplitudes) during trials in which they won the opportunity to react with vengeance by administering noise blasts to opponents who gave them insulting feedback (compared with trials without revenge; Threadgill & Gable, 2020). The authors concluded that anger, a highly intensive negative affective state, appears to promote action tendencies motivated by the pursuit of a goal and enhances reward-related brain activity, particularly in social situations that incite a desire for revenge (Threadgill & Gable, 2020).

The link between reactive aggression and PA can also manifest in intergroup relational contexts. Some of the research investigating psychological processes associated with spontaneous participation in intergroup violence (riots, communal/street fights, rebellions, lynch mobs) suggests that social precursors that render one’s group identity highly salient may contribute to a strong (somewhat self-sacrificial) impetus to pursue a shared ideal or desire (Bostock, 2010; Drury, 2020; Gallo & Economy, 2016; McDoom, 2013). A tangible example of this in seen in Irish faction fighting, whereby, once a communal fight between rival factions began, friends and families, including children and neighboring villagers, would spontaneously react to these events by joining in (Conley, 1999). These collective episodes of violence are thought to have increased PA (at least in part) because of the social rewards they produced. Demonstrating faction loyalty through participation solidified group bonds and afforded opportunities for social mobilization (e.g., improving group status; Conley, 1999). Further, there appear to have been few social or legal restraints on these fighting events because they were deemed largely socially acceptable and rarely implied any legal consequences when innocent bystanders were killed (Conley, 1999).

Research examining mechanisms underlying positive perceptions of intergroup reactive aggression point to several sources of social influence. Although there are a variety of pretenses on which the group is formed (e.g., based on race, ethnicity, political views, team sports affiliations), there are key external sources of social pressure that increase the desire to react aggressively in intergroup contexts. One of the most powerful sources can come from a strong group leader, who will construct, promote, and enhance the salience of the rewarding effects of intergroup violence (Littman & Paluck, 2015). Group leaders reinforce and strengthen identification with the “us” (group identification) by delivering persuading messages and promises that ramp up group emotions and mobilize immediate collective action. For individuals who strongly identify with the group leader’s narrative (prototypical members), demonstrating their loyalty through prescribed action can become a virtuous, positive experience (Reicher et al., 2008). Although obedient action may, in itself, be rewarding in that it can generate a sense of “living up” to group ideals, some leaders provide material rewards for those who jump into action and punish individuals who hesitate or fail to act when the situation arises (Collier & Hoeffler, 2004). Further, prototypical members can incentivize fellow in-group members to participate by supplying additional rewards, such as positive appraisals, recognition, and respect, and act on behalf of the leader by punishing members who do not participate by ensuring they experience shame, rejection, and even physical pain (Littman & Paluck, 2015; Nawata & Yamaguchi, 2013). Further research is needed to examine the role of collective motives in driving increases in PA, specifically in the context of intergroup aggression.

### Proactive forms of PA-related aggression

For some individuals, the thought of experiencing a rush or thrill by inflicting harm on another person can lead them to seek out opportunities to aggress proactively. A desire for “excitement” has been shown to be an important precursor of adolescent violent offenses (Gudjonsson & Sigurdsson, 2007). Boredom, which is often accompanied by feelings of loneliness and negative emotions such as anger, sadness, worry, and despair (Chin et al., 2017), has also been linked to the initiation of harmful, sadistic acts toward others (Pfattheicher et al., 2021). Many theories and studies have centered on trying to explain the role of proactive aggression in relation to PA (or pleasure, excitement, or sensation seeking). We discuss some of the more prominent research while acknowledging the wide range of factors that contribute to these associations.

Several studies have emphasized the role of certain key personality characteristics among the more popular links between proactive aggression and PA. A tendency to disregard, minimize, or lack a shared understanding of another person’s emotional experience—the defining features of reduced empathy—has been suggested as one of the primary commonalities among individual personality trait factors linking proactive aggression, sensation-seeking behavior, and PA (Berkowitz, 1993; Dodge & Coie, 1987; Pajevic et al., 2018). In the classic study by Boldizar et al. (1989), aggressive boys not only considered aggression more personally rewarding than their less aggressive peers but also reported being less concerned about the suffering experienced by their victims, as well as the risk of retaliation, social rejection, or negative self-evaluations. Further, the satisfaction inherent in their aggressive behavior was suggested to derive from having a personal desire to experience control over their victims, as these young aggressors deemed this to be the most valuable aspect of the behavior (Boldizar et al., 1989).

There is also evidence of a distinct set of “dark” personality traits comprising a constellation of antisocial tendencies that are associated with both a lack empathy and a tendency to engage in PA-related proactive forms of aggression (Pajevic et al., 2018). The grandiose subtype of narcissism, for example, is featured by an entitled sense of self and exploitative interpersonal encounters serving to fulfill ego-reinforcement needs (Jones & Paulhus, 2014). Psychopathy, which is characterized by an acute lack of empathy and a desire to seek stimulation and escape from boredom through terrorizing and harming others, may represent another example (Cima & Raine, 2009). Last, and most closely associated with the aggression-pleasure link, is sadism, in which PA is thought to be most directly derived from inflicting harm and deliberately engaging in cruel and vicious behavior “just for the sake of it” (O’Meara et al., 2011). Sadistic tendencies have been uniquely associated with proactive forms of PA-related aggression, such as bullying and Internet trolling (i.e., disruptive, deceptive, destructive online behavior intended to provoke and harm others for amusement; March & Steele, 2020), with Internet trolls reportedly seeking out their victims for personal entertainment (Phillips et al., 2006). The studies showing underlying mood-repair motives in relation to reactive aggression and PA also found that sadistic impulses mediated these effects. Further research is needed to determine the extent to which individual personality such as narcissism, psychopathy, and sadism contribute to the relationship between aggression and PA in different relational contexts (e.g., interpersonal vs. intergroup); however, there does appear to be a wealth of evidence converging on the idea that aggression can effectively elicit PA when there is a lack of empathy for the victim, when it combats negative feelings or thoughts, and when it successfully reinforces positive perceptions of the self.

Within intergroup relational contexts, the relationship between proactive aggression and PA may be understood by considering how collective ideals influence an individual’s emotional experience. For example, certain sports are associated with a positive perception of violence, with athletes who “play dirty” and frequently initiate fights being perceived as fierce, fearless, and strong. The negative consequences (e.g., seriously harming another player, getting a penalty, being suspended) can seemingly be negligible, as aggressive displays of team dominance and strength are often met with praise from coaches, parents, teammates, and supportive fans (Smith, 1979; Wajman, 2017). Even George Orwell (1945) wrote, when referring to violence in competitive sports, “serious sport has nothing to do with fair play” (p. 11). Indeed, these types of relational contexts carry with them a set of social and moral codes that influence how we perceive and interact with others, often promoting the type of proactive aggression that elicits PA on a collective level.

Findings from research on school-bullying groups also point to the reinforcing and rewarding effects of proactive forms of intergroup aggression. “Ringleaders,” or bullying “reinforcers,” provide positive feedback after episodes of group bullying (i.e., an incident of coercive and harmful behavior committed by a group of bullies toward “weaker” individuals; Salmivalli et al., 1996) and incite members to join in with collective praise (e.g., cheering, laughing, giving high fives; Salmivalli, 2010). Repeated acts of collective dominance can reinforce the popularity and prestige of the group, increasing their perceived group status and power over other individuals in the school (for review, see Salmivalli, 2010). Although the degree to which these acts evoke a PA response may vary across group members, there does appear to be a highly influential group effect on individual emotions. More specifically, because an individual’s experience is perceived through the lens of the group, positive appraisals of group behaviors can effectively elicit positive group emotions (Smith et al., 2007).

There is ample supporting evidence indicating an important relationship between proactive intergroup aggression and PA. The highly influential study on violent adult males by Toch (1992) found that 6% of the sample reported experiencing pleasure in bullying and inflicting harm and deliberately sought opportunities to be violent and merciless toward their victims. The classic study by Zimbardo found that a significant minority of participants enjoyed torturing helpless prisoners (Zimbardo et al., 1999). Meyer-Parlapanis et al. (2015) found PA-related aggression increases in conflict regions across all combatants, regardless of whether they are male and female. From combatants of war (Bourke, 1999; Glowacki & Wrangham, 2013; Hatzfeld, 2005; Higgins, 2004; Saramifar, 2019) to members of violent gangs (see Weierstall et al., 2013) and football (soccer) hooligans (Brown & Brittle, 2008; Spaaij, 2008), first-person accounts of collective violence are often associated with PA. In the novel *Villains* (Brown & Brittle, 2008), personal testimonies of former football hooligans describe the PA they experienced during the preaggression phase: “They were all grinning and waiting for their prey to be thrown to the pack. In seconds it started, whack after whack after whack” (p. 147). This type of narrative has been echoed across historical texts and socioeconomical contexts, often accompanied by a reported desire to earn respect, experience a victory, dominate the weak, or destroy the enemy.

### Nontraditional forms of PA-related aggression: indirect, observational, and vicarious

Despite differences in how aggression is defined, there are many ways to engage in notions of “harming others” that can equally elicit PA. Using a multifaceted approach, we can draw from the literature on the appeal of violent media (e.g., film, music, video games), vicarious aggression, and schadenfreude to elucidate instances of PA-related aggression that are perhaps less traditional but more widely shared across cultures and societies. Although terms such as “schadenfreude” (translated literally as “harm joy” from German) and “vicarious aggression” are not as common in the North American aggression literature, research investigating these phenomena, as well as other nontraditional forms of aggression, shows considerable corroborating evidence of a relationship between perceiving the physical or psychological pain of another person (or persons) and experiencing PA in response.

The appeal and popularity of violence in media (music, television, film, video games, etc.) have been extensively researched, with recent studies beginning to place a more concerted focus on PA effects. Engaging in virtual aggression through playing violent games is a “nontraditional” way of increasing PA, at least for some users, with reported cathartic effects, mood improvement, and feelings of happiness, euphoria, and less distress after playing (Bösche, 2010; Grodal, 2000; Kersten & Greitemeyer, 2022; Ravaja et al., 2008). Similarly, music genres characterized by violent content can evoke positive emotions. A recent study investigating differences in affective response after listening to or viewing the lyrics of extremely violent themes depicted in excerpts of death metal (a musical genre often containing extremely violent content; Christenson et al., 2012) found that heavy-metal fans reported significantly higher PA, power, nostalgia, and transcendence than non-heavy-metal fans, as well as significantly less anger and tension (Thompson et al., 2019).

Finally, studies have also shown that simply watching others experience suffering, particularly when they belong to an out-group, can provoke an increase in PA. A study by Cikara et al. (2011) examining occurrences of schadenfreude between fans of two competing baseball teams revealed that witnessing a rival team’s loss while watching video excerpts of a game increased pleasure ratings. Indeed, experiencing schadenfreude, or enjoying virtual or vicarious forms of aggression, may not require one to harm others in the traditional sense; however, the elicited PA actualizes as a real experience for the individual in direct association with aggressive acts.

The role of these nontraditional forms of aggression in increasing PA within interpersonal and intergroup social interactions remains poorly understood despite the fact that these associations often arise in everyday life across most societies. Scholars and researchers have made multiple attempts at theorizing, evaluating, and explaining the positive aspects of aggression, basing their understanding on different theoretical perspectives and often conflicting findings. This article, although only scratching the surface of supporting evidence for the complex relationship between aggression and PA, elucidates the need for a more comprehensive and unified approach to conceptualizing this phenomenon. On the basis of extant theoretical and empirical literature and on an explorational analysis of how they might converge, we address this challenge by proposing a novel, integrative model of PA-related aggression that highlights the role aggression plays in eliciting PA across diverse social situations and relational contexts.

## An Integrative Approach to the Concept of PA-Related Aggression

Current models of PA-related aggression make a significant contribution to our understanding of the phenomenon. However, unlike other types of aggression, these models have not developed to the same extent and remain few. Despite growing interest in researching this relationship, when considering the fundamental theoretical differences across models, it appears that we are not moving forward but rather going in circles. Indeed, in the case of psychopathy and certain personality disorders, the classic viewpoint considers the PA experience sought by carrying out aggressive or violent acts as the primary motivation of aggressive behavior. The evolutionary stance proposes that this motivation is distinct from psychopathy and psychopathology, existing in all human beings who have the potential to feel pleasure by performing violent acts, and provided that these same individuals are placed in a context that is conducive to the emergence of this motivation. Finally, the emotion-regulation argument is based on the premise that all human beings have the potential to use aggressive behaviors as a strategy to feel better after a provocation or social rejection, but this would primarily be the case for individuals who present sadistic traits and use these strategies.

We propose that one of the reasons for the lack of unity among the models stems from the fact that each specifically accounts for a different subtype of PA-related aggression, which leaves little opportunity to build conceptual bridges between them. By proposing four conceptual categories that encompass all manifestations of the phenomenon, it becomes possible to highlight mechanisms potentially common to each category. The four PA-related aggression categories are interpersonal-reactive PA-related aggression, interpersonal-proactive PA-related aggression, intergroup-reactive PA-related aggression, and intergroup-proactive PA-related aggression.

Moreover, the two essential questions that need to be answered to unify current models pertain to how aggression relates to PA and whether it is a universal phenomenon or rather concerns individual traits. First, considering the variety of its forms and the wide range of social contexts in which it occurs, we believe that PA-related aggression is indeed a universal phenomenon, as suggested in the evolutionary model. However, we would also propose that, in parallel with an innate motivation to hunt, kill, and reap PA-eliciting rewards, there would also have been an omnipresent experience of *threat* during these aggressive interactions stemming from having likewise been preyed upon by a predator or from having one’s “catch” stolen by conspecifics. By proposing an association between threat and pleasure, in relation to aggression, the evolutionary model can be combined with the emotion-regulation model, which posits a link between NA and PA. Second, regarding the development of individual differences in PA response to aggression, we posit that the threat-reward cycle is maintained according to the individual’s personal history and that this would facilitate passage from primarily reactive to proactive functions of the behavior. Let us consider this theoretical model in more detail and see how it fits when applying our four categories.

First, the emotion-regulation model (e.g., Chester, 2017) can be conceptualized as specifically addressing behaviors grouped under the interpersonal-reactive category of PA-related aggression. This model proposes that the affective response associated with aggression occurs sequentially, with NA occurring during the preaggression phase and PA occurring during or after the act. Specifically, the NA (e.g., anger, shame) resulting from a social provocation or rejection event motivates the individual to use aggression to repair a negative mood and obtain pleasure from getting revenge. Interpersonal contexts involving social provocations can indeed lead to acts of aggression, albeit to varying degrees of severity. Nevertheless, these acts can cause the other person (the offender) suffering and pain. The pleasure felt by causing this suffering, the psychological mechanism responsible for mood repair, is particularly associated with sadistic traits (Chester et al., 2019). However, there is evidence in the literature of other personality traits that can lead an individual to aggress in socially threatening contexts, such as narcissism (Baumeister & Campbell, 1999) and psychopathy (Cale & Lilienfeld, 2006), although it has yet to be demonstrated whether these traits are specifically associated with a PA response in the context of aggression. If this were indeed the case, these personality traits could potentially be associated with different types of social threats, and the PA-related aggression would be associated with the removal of these threats. For example, the PA response occurs while hurting another person for the sadist, after humiliating them for the narcissist, and after dominating them for the psychopath. In all cases, these manifestations of PA-related aggression are characterized by the presence of a social threat likely to evoke NA that is effectively “repaired” via the PA evoked through aggression.

In our interpersonal-proactive category of PA-related aggression, we find a more classic model of appetitive aggression (Meloy, 2006; Williamson et al., 1987), here stipulating that there are individuals with pathological personality traits who seek to aggress for pleasure or arousal. If we consider psychopathy and other forms of personality disorder, for example, aberrant motivations can manifest in different contexts. The actions of these individuals can be conceived as occurring on a continuum of severity ranging from mild to the most violent forms of aggression. There is evidence that certain personality disorders are associated with a history of physical and/or psychological abuse (Shi et al., 2012). Thus, it is possible that, in accordance with the emotion-regulation model, the PA experiences of an individual proactively aggressing to feel pleasure have already been associated in the past with repeated social threats eliciting NA. On the basis of this personal history, the individual may have been led to develop strategies of using aggression to repair negative affective states. In such a case, we theorize that an associative learning between a social threat and the pleasure of aggressing is formed over the course of one’s life history, characterized by multiple events of more or less intense provocations occurring in certain social contexts (e.g., at home, in school, with police/correctional officers). Accordingly, when placed in similar contexts, the individual would experience an implicit (or unconscious) threat accompanied by an explicit (or conscious) anticipation of the pleasure obtained through aggression. Over the course of the individual’s development, there would thus be a shift from the reactive to the proactive function of aggression, and the threat–pleasure sequence would cease to be cyclical (as is the case with the interpersonal-reactive category) but rather maintained over time through seeking out or being exposed to similar threatening contexts. In summary, the interpersonal-proactive category would be characterized by an anticipation of reward (PA), whereby the desire for aggression is fueled by the threat elicited in certain social contexts.

Elbert’s (2017a) appetitive-aggression model falls more naturally into the proactive-intergroup PA-related aggression category. The model describes the rewarding (PA) experience of engaging in violent acts in the context of combat. Weierstall and Elbert (2011) developed a scale, the Appetitive Aggression Scale, that performs well in assessing an individual’s desire to perform potentially lethal acts in a group context. It is interesting to note that, according to the authors, the pleasure is not instantaneous. Individuals who report engaging in appetitive aggression often describe an initial period of anguish and horror (see Elbert et al., 2010). It is thus possible, here again, to theorize that the PA response elicited during an act of violence is accompanied by an equally acute threat. When the individual remains in a combat context in which the threat is continually present, the association between the implicitly experienced threat and the explicitly anticipated reward would maintain this cycle of repair. It is also possible that the greater the threat (e.g., when facing life-threatening situations), the greater the elicited PA. The association could develop after a single incident that is highly intense or after repeated engagement in high-risk situations, and the pleasure experienced when “eliminating” the source of the threat (i.e., the person or persons attacking) may result in an increase of trait appetitive aggressiveness that can manifest in postcombat contexts. Further, although the enemy is easily detectible in situations of combat, any intergroup context potentially poses a threat when a member of an out-group is encountered. The out-group serves as the object of threat-related implicit cognitive attributions (i.e., ascribing qualities to others outside conscious awareness) that may be reinforced by other social processes and biases, such as dehumanization, stereotypes, racism, or prejudice. In sum, our proposed proactive-intergroup category is characterized by (potentially) extreme manifestations of PA resulting from having survived equally extreme threats associated with acts of violence. The violent nature of these behaviors can be explained by the resources available in these situations (e.g., organization, weapons) and by the influence of social thought processes often emerging in intergroup contexts (e.g., social biases).

For our last category, reactive-intergroup PA-related aggression, there does not appear to be a specific model that aptly explains it. However, in keeping with the argument that a cycle promotes passage from reactive to proactive functions of the behavior, we can conceive of behaviors in this category as representing the original source of those encountered in the proactive-intergroup category. The difference here, again, is that the cycle does not continue; after an incident of being attacked, and experiencing the pleasure of surviving the attack, this association is never reinforced. For example, this category might include the individual who used aggression to fight some form of threat (e.g., social, risk of death) in an intergroup context, the elicited PA effectively repaired the threat to their group identity, but circumstantial changes in their life discouraged or prevented this association from being sustained. One can imagine a soldier who receives a military discharge because of an injury or a criminal offender who receives rehabilitation support. The threat–pleasure sequence would likely not have sufficiently been repeated for the function of aggression to shift from the reactive to the proactive domain.

Overall, we can conclude that the transition from the reactive to the proactive category is facilitated through solidification of the associations made between the threat and the reward (PA response), accompanied by anticipation of the reward, which would favor the maintenance of the cycle in different contexts. Differences between interpersonal and intergroup relational contexts largely pertain to the severity of the aggressive acts, which will depend on situation-specific affordances (e.g., available resources), and the influence of implicit social thought processes. Further, in all four categories of PA-related aggression, behavioral manifestations would be facilitated by social and psychological processes that lead to a reduction of empathy toward the victim or their associated group. In interpersonal contexts (for both reactive and proactive aggression), personality traits characterized by a lack of empathy, having a tendency to use or abuse substances, or presenting a deficit in impulse control would all reduce an individual’s ability to consider the victim’s pain. In intergroup contexts, processes that lead to the disempowerment of perpetrators (Hodson et al., 2014), or the dehumanization of victims (see Haslam, 2006), would allow the individual to protect themselves from the trauma associated with the cruelty of their actions (Weierstall & Elbert, 2011).

Although virtual and vicarious forms of harmful behavior are not generally recognized as actual forms of aggression, their inclusion in our integrative model seems particularly important because they provide support for the theory that this phenomenon is universal. The pleasure evoked from witnessing one’s sports team “butcher” the rivals, or while watching a fight between hockey players or boxers, or from watching a film about vengeance all represent the types of PA-related aggression commonly experienced by all human beings, even if at varying degrees of intensity. Scholars have proposed different theoretical explanations for why our capacity for empathy toward the victim can seemingly be momentarily extinguished in certain social situations, as well as why the aggressive actions of another person can elicit a PA response in ourselves. One possibility is that, because the aggression is fictional, or socially sanctioned, or not committed by us, we can set aside empathetic concern and moral constraints and simply give in to our innate, pleasure-evoking, aggressive urges. The degree to which these forms of aggression elicit PA may depend on these temporary suspensions of everyday conventions, thus reducing any risk of perpetrator remorse or feelings of guilt (for a review specific to media violence, see Hartmann & Vorderer, 2010). The degree to which we perceive the victim(s) as fully human is another important factor to consider because processes of dehumanization have been shown to down-regulate the capacity for compassion and empathy (Jhurry & Harris, 2021), which is likely further down-regulated in contexts of virtual aggression.

The classic “uses-and-gratifications” theory (Blumler & Katz, 1974), a model of the appeal of media in fulfilling certain fundamental psychological needs (e.g., autonomy, competence, relatedness), suggests that the appeal of virtual violence lies in its ability to provide users with opportunities to experience power and domination as they successfully defeat others and feel effective in their efforts (Grodal, 2000; Klimmt et al., 2006; Sherry, 2004). In addition, by blurring the boundaries between fiction and reality, unresolved real-life conflicts can be redirected and faced without inhibition (Zillmann, 1998), allowing the individual to vent out their frustrations and eliminate real threats symbolically (through identification with one’s virtual self), which would have liberating, cathartic, indeed PA-eliciting effects. With vicarious forms of aggression, which allows individuals to experience PA-related aggression through the actions of another person (e.g., an athlete, character in a film), conventional constraints are similarly suspended, allowing the individual to experience the rewarding effects of having eliminated a perceived threat.

Although these explanations have some empirical backing, they are largely tentative and exploratory in nature. Ultimately, they point to the relevance of considering at least two psychological mechanisms that would operate together to explain how virtual and vicarious forms of aggression have the potential to elicit PA across all individuals. They comprise *symbolic thinking* (e.g., the cognitive process whereby one chooses to perceive fictional events as real) and *identification* (e.g., the psychological process in which an individual has experiences vicariously through the actions of another person) and would serve as instrumental capabilities that make it possible to experience PA in both virtual and variant modes. Other dimensions contributing to PA effects could include the active versus passive and/or first-person versus observer nature of the aggressive interaction. In any case, research is needed to better identify the types of individual differences that explain why one individual feels satisfied when engaging aggressively in the virtual realm, whereas others require actual or both (real and virtual) to achieve similar effects.

## Conclusion and Future Directions

The purpose of this article was to reinvigorate the concept of PA-related aggression by reviewing and organizing theories and empirical findings. On the basis of existing literature, we further proposed an integrative model to generate testable hypotheses and help direct future research incentives. Specifically, we proposed that empirical investigations at the level of interpersonal relational contexts would benefit from focusing efforts on the function of proactive forms of PA-related aggression, particularly in relation to addictive and/or chronic manifestations of the behavior. Our personal-history hypothesis, which posits that an associative learning occurs between repeated experiences of social threat and PA-related aggression, could be tested cross-sectionally or longitudinally by studying the links between sadistic traits, a history of abuse, and PA-related aggression. Our hypothesis of anticipated threat within certain specific social contexts could be tested experimentally by exposing participants with personality disorders (vs. controls) to conditions evoking a threat without actual provocation, allowing the unique effects of threat on PA-related aggression to be assessed. At the level of intergroup relational context, it would be interesting to compare individuals who experience PA-related when identifying with their group (on an occasional basis) with those who display it chronically. This would allow us to better understand not only the threshold at which aggression evokes PA but also the threshold at which reactive manifestations shift toward proactive tendencies. Finally, the moderating factors of all PA-related categories require further research. Among these factors, the capacity for symbolic thought is of particular interest because it may help us better understand its role in virtual and vicarious aggression-related PA and bring us closer to explaining why it possibly replaces the effects of actual (real-world) manifestations of the phenomena.

This theoretical model needs to be validated before it can be applied to prevention and treatment efforts. Nevertheless, we can already envision how the model can serve to inform prevention programs that target the threat-reward (PA response) cycle of violence during child development, the cycle of violence between social groups, and identification processes activated within social contexts of threat toward individuals habitually manifesting PA-related aggression. Prevention techniques involving training in the generation of alternative interpretations or behavioral responses to perceived threats may help these individuals manage future (perceived) threats in an adaptive, nonaggressive, and less harmful manner.

## Transparency

*Action Editor:* Leonel Garcia-Marques

*Editor:* Interim Editorial Panel

*Author Contributions*

J. E. Quansah conceptualized the initial objectives, content, function, and structure of the manuscript, researched the literature, and wrote and revised the original draft. J. Gagnon conceptualized and wrote the theoretical section, which was revised by J. E. Quansah. Both authors approved the final manuscript for submission.
